# Role of influenza vaccine during pregnancy in protecting against pertussis

**DOI:** 10.1056/NEJMc1705208

**Published:** 2018-03-29

**Authors:** Marta C. Nunes, Clare L. Cutland, Shabir A. Madhi

**Affiliations:** 1 Department of Science and Technology/National Research Foundation: Vaccine Preventable Diseases, University of the Witwatersrand, Johannesburg, South Africa; 2 Medical Research Council: Respiratory and Meningeal Pathogens Research Unit, University of the Witwatersrand, Johannesburg, South Africa; 3 National Institute for Communicable Diseases: a division of National Health Laboratory Service, Centre for Vaccines and Immunology, Johannesburg, South Africa

Evidence from epidemiological studies and challenge experiments in animal models have demonstrated that influenza virus infection can enhance the susceptibility to infection by bacteria, such as *Streptococcus, Haemophilus influenzae* and *Staphylococcus aureus*^1^. The relationship between influenza virus and atypical bacteria, namely *Bordetella pertussis* has, however not been well characterized^2^.

In a randomized, double-blind, placebo-controlled trial conducted in South Africa in 2011 and 2012 we have shown that influenza vaccination of HIV-uninfected pregnant women was 50.4% efficacious in preventing polymerase-chain-reaction (PCR) confirmed influenza infection from the time of enrolment to 24-weeks post-partum^3^. Here we present the results from the retrospective testing by PCR for *B. pertussis* infection of the maternal pharyngeal specimens collected at the time of respiratory illnesses. The PCR protocol and results interpretation have been described^4^.

Overall 2116 women were enrolled in the study including 1062 in the influenza vaccine- group and 1054 in the placebo-group. A total of 3583 respiratory specimens were collected from 1361 participants from enrolment to 24-weeks post-partum, and of these 3125 (87.2%) specimens were tested for *B. pertussis*. Eleven vaccine-recipients tested pertussis PCR- positive compared to 26 placebo-recipients (risk-ratio 0.4 [95%CI: 0.2, 0.8]). Further, there were 10 and 16 women in the vaccine and placebo groups, respectively, who were pertussis PCR-indeterminate on testing, Table. The overall risk-ratio, including the PCR-indeterminate episodes was 0.5 (95%CI: 0.2, 0.7). Thirty-three pertussis episodes among the women occurred post-delivery, with infants testing pertussis PCR-positive at the sometime as the mother on five occasions (three among the vaccine-group), within 22-days of maternal episode on two occasions (both in the placebo-group), and between 52 to 73 days after the maternal episode on three occasions (all in the placebo-group). No pertussis episodes were observed in the mothers a posteriori from the infants’.

**Table:** Detection rate of *Bordetella pertussis* in women who participated in a randomized, double-blind, placebo-controlled trial of trivalent inactivated influenza vaccine 95%CI: 95% confidence interval; PCR: polymerase chain reaction. Archived DNA samples independently of clinical presentation were tested by real-time PCR for the presence of the multicopy pertussis insertion sequence (IS) *IS481*^4^; if *IS481* cycle threshold values were <40, total nucleic acids were extracted from the corresponding achieved respiratory specimen using a NucliSENS easyMAG (bioMerieux) platform and re-tested for *IS481* and in a duplex reaction for *hIS1001* and *pIS1001*, and in a singleplex reaction for the pertussis toxin subunit S1 (*ptx*S1). Primers and probes used, and PCR results interpretation have been described^4^.

	Influenza vaccine N=1062^a^	Placebo N=1054^a^	Risk-Ratio (95%CI)	P value
**Participants with at least one specimen collected, no (%)**^b^	675 (63.6)	686 (65.1)	1.0 (0.9, 1.0)	0.46
**Participants with at least one specimen tested by PCR for pertussis, no (%)**^b^	635 (59.8)	652 (61.9)	1.0 (0.9, 1.0)	0.33
**Respiratory specimens collected**	1713	1870	-	-
**Respiratory specimens tested by PCR for pertussis, no (%)**^c^	1494 (87.2)	1631 (87.2)	1.0 (0.9, 1.0)	0.99
**Pertussis PCR-positive cases, no (%)**^d^	11 (1.0)^f^	26 (2.5)^g^	0.4 (0.2, 0.8)	0.012
**Pertussis PCR-indeterminate cases, no (%)**^e^	10 (0.9)	16 (1.5)	0.6 (0.3, 1.4)	0.23
**Overall pertussis cases, no (%)**	21 (2.0)	42 (4.0)	0.5 (0.2, 0.7)	0.007

a Participants were followed-up by weekly active surveillance for any respiratory illness from the time of enrolment through pregnancy to 24- weeks post-partum. Pharyngeal specimens were collected by study staff at the time of respiratory illness visits to the study clinic.

b Percentage calculated from total participants enrolled. Respiratory specimens only collected in participants presenting with a respiratory illness.

c Percentage calculated from total specimens collected.

d PCR reaction with cycle threshold values for the *IS481* gene <35 or ≤35-<40 plus positive for the *ptx*S1 gene.

e PCR reaction with cycle threshold values for the *IS481* gene ≤35-<40 and negative for the *ptx*S1 gene.

f One specimen also tested positive for influenza A/H1N1.

g Two specimens also tested positive for influenza A/H3N2. One participant tested pertussis PCR-positive in two different specimens collected 22 days apart, only the first episode was included.

Our results suggest that influenza vaccination had a protective impact in the rates of *B. pertussis* infection in adult women. This novel observation deserves further investigation on the possible mechanisms in the upper-respiratory tract that can lead to the synergy between the two pathogens. Also, the possible impact of vaccinating mothers against influenza in the transmission of *B. pertussis* to their young infants warrants further consideration, as most severe pertussis disease occurs prior to them completing their immunization against pertussis, and household contacts, particularly mothers have been identified as major sources of infection to the infants^5^. Combined influenza and pertussis vaccination during pregnancy might have a cumulative benefit against *B. pertussis* infection.
